# Root-associated entomopathogenic fungi manipulate host plants to attract herbivorous insects

**DOI:** 10.1038/s41598-020-80123-5

**Published:** 2020-12-30

**Authors:** Belén Cotes, Gunda Thöming, Carol V. Amaya-Gómez, Ondřej Novák, Christian Nansen

**Affiliations:** 1 Integrated Plant Protection Unit, Department of Plant Protection Biology, Swedish University of Agricultural Sciences, 230 53 Alnarp, Sweden; 2 Division for Biotechnology and Plant Health, Norwegian Institute of Bioeconomy Research, 1433 Ås, Norway; 3 Corporación Colombiana de Investigación Agropecuaria (AGROSAVIA), La Libertad, 900005 Villavicencio, Colombia; 4 Laboratory of Growth Regulators, Faculty of Science, Palacký University and Institute of Experimental Botany, The Czech Academy of Sciences, 78371 Olomouc, Czech Republic; 5 Department of Entomology and Nematology, University of California, Davis, CA 95616 USA

**Keywords:** Zoology, Entomology, Plant sciences, Plant physiology, Microbiology, Fungi, Ecology, Behavioural ecology

## Abstract

Root-associated entomopathogenic fungi (R-AEF) indirectly influence herbivorous insect performance. However, host plant-R-AEF interactions and R-AEF as biological control agents have been studied independently and without much attention to the potential synergy between these functional traits. In this study, we evaluated behavioral responses of cabbage root flies [*Delia radicum* L. (Diptera: Anthomyiidae)] to a host plant (white cabbage cabbage *Brassica oleracea* var. *capitata* f. *alba* cv. Castello L.) with and without the R-AEF *Metarhizium brunneum* (Petch). We performed experiments on leaf reflectance, phytohormonal composition and host plant location behavior (behavioral processes that contribute to locating and selecting an adequate host plant in the environment). Compared to control host plants, R-AEF inoculation caused, on one hand, a decrease in reflectance of host plant leaves in the near-infrared portion of the radiometric spectrum and, on the other, an increase in the production of jasmonic, (+)-7-iso-jasmonoyl-l-isoleucine and salicylic acid in certain parts of the host plant. Under both greenhouse and field settings, landing and oviposition by cabbage root fly females were positively affected by R-AEF inoculation of host plants. The fungal-induced change in leaf reflectance may have altered visual cues used by the cabbage root flies in their host plant selection. This is the first study providing evidence for the hypothesis that R-AEF manipulate the suitability of their host plant to attract herbivorous insects.

## Introduction

Biological interactions between plants and root-inhabiting fungi have been found in the fossil record^1^, implying a symbiotic success story. Furthermore, no plant species is known to grow successfully without a relationship with fungi^2^. Root-associated fungi can colonize both the internal and external parts of developing roots without damaging the host plant^3^. The presence of root inhabiting fungi is associated with enhancements in plant growth, i.e. fungal endophytes facilitate host uptake of soil nutrients and water^4^. Concurrently with functional research into complex ecological interactions between plant-associated fungi, their host plants and plant-associated insects there are also applied studies describing the use of multiple strains of *Beauveria* spp. and *Metarhizium* spp. (Hypocreales: Ascomycota) as root-associated entomopathogenic fungi (R-AEF)^5^. R-AEF can be plant endophytes and soil saprophytes, and some have been reported to reduce herbivore damage by direct insect infection and are commercially available for arthropod pest management in a wide range of cropping systems^6^. In addition, R-AEF have been shown to increase host plant resistance to arthropod herbivores by triggering plant induced systemic resistance or to produce secondary metabolites that elicit feeding deterrence and antibiosis to herbivorous insect^6^. When R-AEF are considered to act as bodyguards, host plants are believed to manipulate insect pathogens to increase their own fitness^7^. However, this concept remains purely speculative and more-complex interactions involving R-AEF and host plants can be expected, mainly due to the inherent risks in terms of increasing herbivorous insect attack^8,9^.

A number of viruses, fungi and bacteria have been shown to maximize recruitment of insect vectors as part of maximizing dispersal to non-infected host plants^10^. However, research into entomopathogenic-fungus-colonized plants and their putative ability to affect animal behavior, remains an incipient field. Manipulations of host organisms (both animal- and plant-pathogen systems) range from changes of host quality traits, such as, improving the nutritional composition of hosts, to changes in traits that influence host plant selection^11^. For instance, manipulations of host plants by insect-pathogens can lead to increased attractiveness to herbivorous insects by inducing behavioral alterations as compared to healthy host plants, which is known as the “fatal attraction phenomenon”^12^. In other cases, herbivorous insects are manipulated by alterations of host plant volatiles and host plant quality to prefer nutritionally sub-optimal host plants of inferior quality, which has been termed the “deceptive host phenotype hypothesis”^12^.

**Figure 1:**
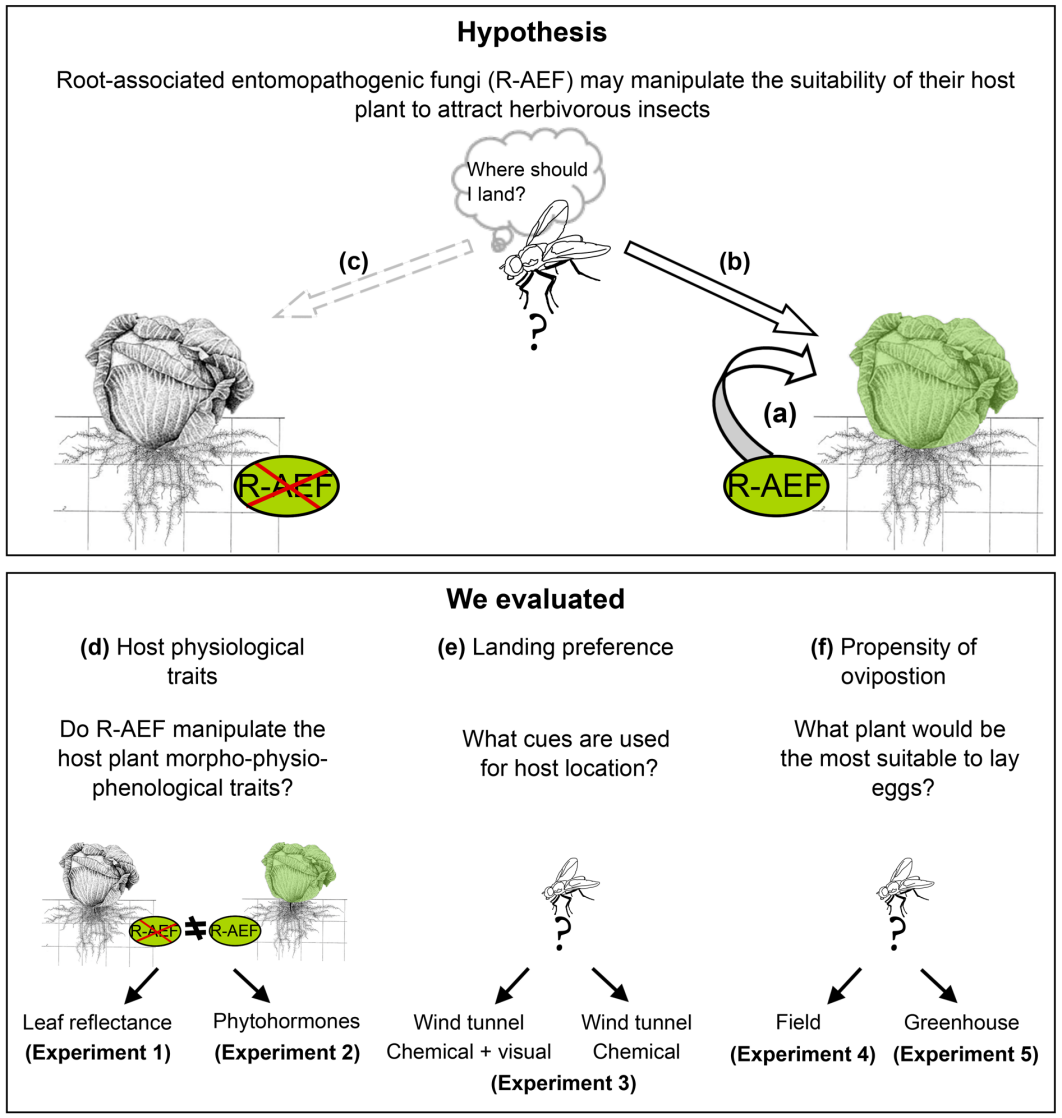
Overview of the research study. The upper panel shows a hypothetical framework describing the effects of the root-associated entomopathogenic fungus (R-AEF) *Metarhizium brunneum* on the behavior of cabbage root flies, *Delia radicum*. R-AEF manipulates the morpho-physiophenological host plant traits by: (**a**) altering leaf reflectance and phytohormonal composition of host plant and (**b**) consequently causing host plants with R-AEF to be more attractive to cabbage root flies than (**c**) host plants without R-AEF. We hypothesized that R-AEF *M. brunneum* manipulates host plants to attract cabbage root flies and increase their dispersal rates. Lower panel shows the sequence of experiments performed to verify the hypothesis. We evaluated the effects of the R-AEF on: (**d**) host plant physiological traits (reflectance and phytohormone measurements) (**e**) landing preference (wind tunnel experiments) and (**f**) propensity of oviposition (field and greenhouse experiments).

In this study, we describe a so far undocumented type of host plant manipulation by micro-organisms. Moreover, R-AEF may increase attractiveness of host plants to healthy herbivorous insects, so that host plants are essentially manipulated by R-AEF to attract and infect insects (Fig. 1a,b). Consequently, we hypothesized that both propensity of landing and oviposition by cabbage root fly females [*Delia radicum* females L. (Diptera: Anthomyiidae)] on R-AEF inoculated host plants (white cabbage *Brassica oleracea* var. *capitata* f. *alba* cv. Castello L.) are significantly increased compared to non-inoculated plants. To address this hypothesis, we performed greenhouse and field experiments with cabbage root flies host plants with and without the bioinsectide Met52 Granular (Novozymes Biologicals Inc., Salem, VA) (Fig. 1d–f). This commercial product is the *M. anisopliae* Strain F52, re-classified as *Metarhizium brunneum* (Petch). Previous studies indicated that the *M. anisopliae* Strain F52 displayed high pathogenicity to cabbage root flies^13^. The cabbage root fly is a major pest of many Brassica crops in temperate regions of Europe and North America. Moreover, cabbage root flies have the ability to pass inoculum of entomopathogenic fungi to other flies^14^, representing an experimental insect model for dispersal of entomopathogenic fungi. More specifically, we investigated whether R-AEF inoculation affects: (1) phytohormonal composition [jasmonic, (+)-7-iso-jasmonoyl-l-isoleucine and salicylic acid] of host plants (and therefore relative suitability of host plants for cabbage root flies), (2) leaf reflectance (and therefore visual cues used by cabbage root flies in their host selection) (Fig. 1d), and (3) host plant location or acceptance behavior of cabbage root flies (Fig. 1e,f).

## Results

### Effects of R-AEF on host plant physiological traits

**Figure 2:**
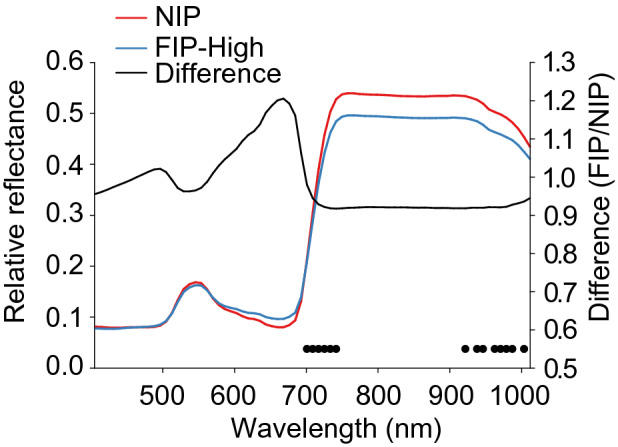
Average leaf reflectance from non-inoculated control plants (NIP) and root-associated entomopathogenic fungal (R-AEF)-inoculated plants at high concentration of fungal inoculum (FIP-High), and the “difference”. Black dots indicate spectral bands, in which average reflectance varied significantly between the two treatments (p < 0.05).

Figure 2 shows the average leaf reflectance from non-inoculated plants (NIP) and fungal-inoculated plants at a high concentration (FIP-High), and the “difference” (FIP/NIP) shows the relative difference between the two host plant treatments. Inoculation caused an increase in leaf reflectance in spectral bands between 550 and 700 nm and a decrease in leaf reflectance in spectral bands between 700 and 1030 nm. Based on analyses of variance of average leaf reflectance in individual spectral bands, we found significant treatment effects (p < 0.05) in individual spectral bands two regions in the near-infrared spectrum: 700–742 nm and 921–1004 nm (indicated by black dots).

**Figure 3:**
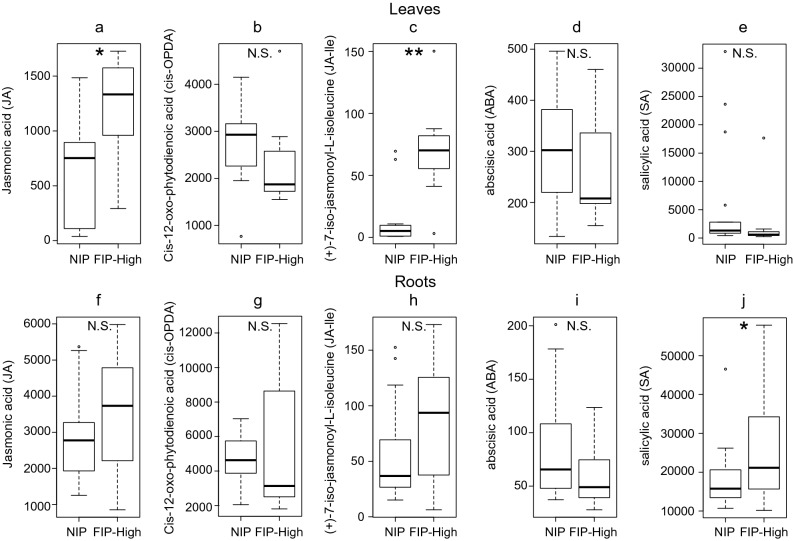
Levels of phytohormones (pmol/g DW) measured in leaves and roots of non-inoculated control plants (NIP) and root-associated entomopathogenic fungal (R-AEF)-inoculated plants at high concentration of fungal inoculum (FIP-High). Boxplots represent phytohormone relative abundances of jasmonic acid (JA), (+)-7-iso-jasmonoyl-l-isoleucine (JA-Ile), cis-12-oxo-phytodienoic acid (cis-OPDA), abscisic acid (ABA) and salicylic acid (SA) measured in leaves (**a**–**e**) and in root sections (**f**–**j**). Boxplots visualize five summary statistics (the middle bar = median; box = inter-quartile range; bars extend to 1.5× the inter-quartile range the median). Stars represent *p < 0.05, **p < 0.01, ***p < 0.001, *N.S.* not significant.

Levels of jasmonic acid (JA), (+)-7-iso-jasmonoyl-l-isoleucine (JA-Ile), cis-12-oxo-phytodienoic acid (cis-OPDA), abscisic acid (ABA) and salicylic acid (SA) measured in host plants leaves and roots for NIP and FIP-High are represented in Fig. 3. In root sections of host plants, SA was significantly higher in FIP-High compared to NIP (Table 1, Fig. 3j). Both JA and JA-Ile were found in higher amounts in the FIP compared to NIP in host plant leaves (Table 1, Fig. 3a,c).

**Table 1:** Summary of models of Experiment 2 showing type of test and distribution, used and results for the fixed factors.

Type of analysis	Statistical test response variable ∼ distribution		Fixed factors	χ^2^	df	p-value
Hormone profiling In leaves	GLM Amount of hormone ∼ Gamma	Jasmonic acid (JA)	Treatment	5.1	1	0.023
		(+)-7-iso-jasmonoyl-l-isoleucine (JA-Ile)	Treatment	7.4	1	0.007
		cis-12-oxo-phytodienoic acid (cis-OPDA)	Treatment	0.9	1	0.347
		Abscisic acid (ABA)	Treatment	0.4	1	0.552
		Salicylic acid (SA)	Treatment	0.5	1	0.477
Hormone profiling In roots	GLM Amount of hormone ∼ Gamma	Jasmonic acid (JA)	Treatment	1.3	1	0.245
		(+)-7-iso-jasmonoyl-l-isoleucine (JA-Ile)	Treatment	3.0	1	0.082
		cis-12-oxo-phytodienoic acid (cis-OPDA)	Treatment	0.6	1	0.427
		Abscisic acid (ABA)	Treatment	2.9	1	0.091
		Salicylic acid (SA)	Treatment	4.5	1	0.033

### Effects of R-AEF on landing preference

**Figure 4:**
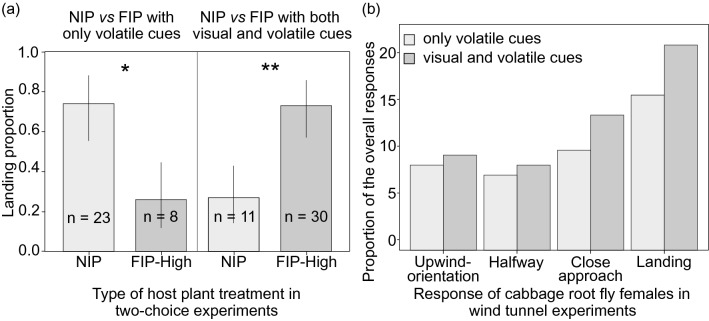
(**a**) Percentage (± coefficient interval) of wind tunnel landings of cabbage root fly females offered non-inoculated control plants (NIP) and root-associated entomopathogenic fungal (R-AEF)-inoculated plants at high concentration of fungal inoculum (FIP-High) in a two-choice wind tunnel experiment encountering only volatile cues and with both visual and volatile cues of host plants*.* Stars represent *p < 0.05, **p < 0.01, ***p < 0.001, *N.S.* not significant. (**b**) Bar plot of the proportion of the overall cabbage root fly females’ responses in a two-choice wind tunnel experiment with only volatile and both volatile and visual stimuli of host plants.

Landing preference of the cabbage root fly (Fig. 1e) was evaluated in a two-choice wind-tunnel experiment (Experiment 3). In presence of volatile cues only, cabbage root fly females performed significantly higher numbers of landings in the NIP compared to FIP-High (t-test, p = 0.011) (Fig. 4a). The opposite response by cabbage root flies was observed testing volatile and visual cues together (whole host plants). The use of a visual cues in addition to odor cues increased the number of landings by cabbage root fly females from 16.6 to 21.9%. Here, a significant higher number of cabbage root fly females landed on the FIP-High compared to NIP (t-test, p = 0.004) (Fig. 4a). For all sequential behavioral steps, the percentage of responding cabbage root flies testing volatiles only and testing volatile and visual cues together are shown in Fig. 4b.

### Effects of R-AEF on propensity of oviposition

**Figure 5:**
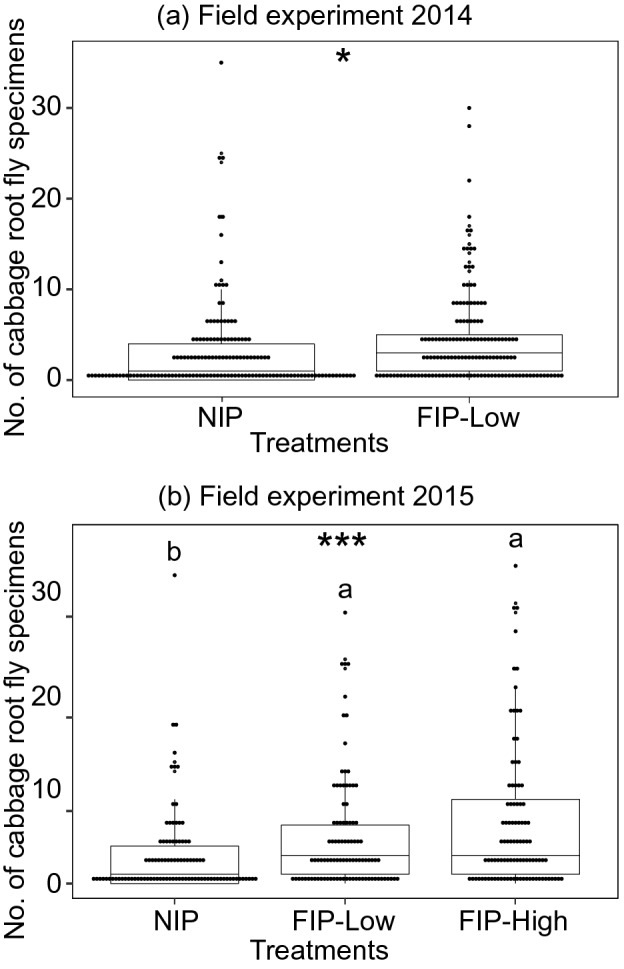
Symmetrical dot-density plot with superimposed box plot showing the distribution of the total number of cabbage root fly specimens collected in non-inoculated control plants (NIP) and root-associated entomopathogenic fungal (R-AEF)-inoculated plants at low (FIP-Low) and high concentrations of fungal inoculum (FIP-High) in (**a**) 2014 and (**b**) 2015. Dots represent individual sampled host plants. Boxplots visualize five summary statistics (the middle bar = median; box = inter-quartile range; bars extend to 1.5× the inter-quartile range the median). Stars represent *p < 0.05, **p < 0.01, ***p < 0.001, *N.S.* not significant. Letters above points indicate significant differences between types of treatments (Tukey’s test: p < 0.05).

Propensity of oviposition by the cabbage root flies (Fig. 1f) was evaluated in field and greenhouse conditions (Experiment 4 and 5). In field experiments, numbers of cabbage root fly larvae and pupae collected from host plants were significantly higher in FIP compared to NIP in both field seasons (Fig. 5) (2014 field season Negative binomial (NB) GLM: χ^2^ = 5.4, d.f. = 1, p < 0.05 and 2015 field season NB GLM: χ^2^ = 21.9, d.f. = 2, p < 0.0001). However in 2015, we found no significant differences between the high and low fungal concentrations. Additionally, we observed a significant effect of the block in 2015 (NB GLM: χ^2^ = 21.3, d.f. = 2, p < 0.0001), but not in 2014 (NB GLM: χ^2^ = 4.0, d.f. = 2, p = 0.138). This significance was due to the fact that in one of the three blocks the mean response was substantially different compared to the other two blocks. Mycosis was not observed on cabbage root fly larvae and pupae recovered from NIPs, but it was observed on larvae and pupae recovered from the FIPs in the field experiment (3.3 ± 1.1%).

**Figure 6:**
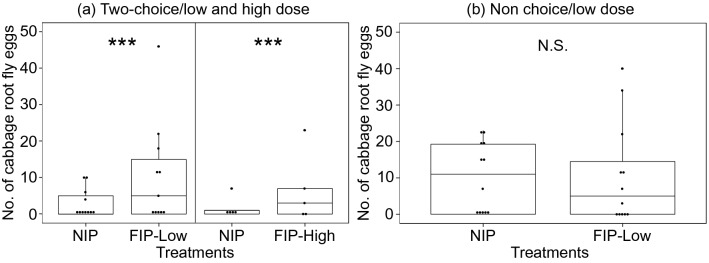
Symmetrical dot-density plot with superimposed box plot showing the distribution of the total number of eggs laid by cabbage root fly females: (**a**) two-choice experiments on non-inoculated control plants (NIP) and root-associated entomopathogenic fungal (R-AEF)-inoculated plants at low (FIP-Low) and high concentration of fungal inoculum (FIP-High) and (**b**) no-choice experiments on NIP and FIP-Low. Dots represent individual sampled host plants. Boxplots visualize five summary statistics (the middle bar = median; box = inter-quartile range; bars extend to 1.5× the inter-quartile range the median). Stars represent *p < 0.05, **p < 0.01, ***p < 0.001, *N.S.* not significant.

Under greenhouse conditions for the two-choice oviposition experiment, a total number of 48 cabbage root fly females were tested, but only 14 cabbage root fly females laid eggs on one of the two host plants and 2 cabbage root fly females on both which were excluded from the preference analysis. The number of eggs was significantly lower on NIP, when these host plants were paired with FIP-Low (Poisson GLMM: Z = 42.8, d.f. = 1, p < 0.001) and with FIP-High (Poisson GLMM: Z = 12.4, d.f. = 1, p < 0.001) (Fig. 6a). In the no-choice oviposition experiment, a total number of 15 out of 24 cabbage root fly females laid eggs on 8 NIP and 7 FIP. No significant differences were observed between host plant treatments (Poisson GLMM: Z = 0.3, d.f. = 1, p = 0.588) (Fig. 6b).

## Discussion

Entomopathogenic fungi face distinct challenges of dispersal and of finding suitable habitats and hosts, and it seems reasonable to assume that strong evolutionary selection pressures are driving manipulative strategies. Such strategies include complex life cycles that include soil, plants, and arthropods as hosts^15–18^. This study provides a novel insight into tritrophic interactions by revealing a novel mode of transmission between R-AEF and herbivorous insects using host plants as intermediate vectors. Moreover, we find argue that some entomopathogenic fungi may be manipulating host plants by increasing their attractiveness to herbivorous insects. We demonstrated that leaf reflectance and phytohormone profiles were altered in host plants in response to R-AEF *M. brunneum* (Fig. 1a,d). These significant changes in host plants aligned with significant increases in propensity of landings and oviposition by cabbage root fly females (Fig. 1b,c,e).

### Direct effects of R-AEF on host plant

R-AEF inoculation caused a significant decrease in average reflectance in two spectral regions in the near-infrared spectrum: 700–742 nm and 921–1004 nm. The 700–742 nm corresponds to the “red edge”, which is caused by the combined effects of strong chlorophyll absorption and leaf internal scattering^19^. The red edge is well known as spectral region to respond to both abiotic and biotic stressors^20^. Several studies have demonstrated that inoculation of plants with pathogenic fungi cause a decrease in leaf reflectance in spectral bands between 900 and 1000 nm^21,22^. In general, average leaf reflectance in spectral bands from 760 to 2500 nm are known to be influenced by factors, such as, leaf structure, water content, surface roughness, and stoma activity^23,24^. Thus, leaf reflectance results corroborate published findings, as the two spectral regions that responded significantly to R-AEF inoculation of cabbage plants are known to be highly responsive and indicative of overall plant health.

R-AEF have been shown to be both plant growth promoters and to increase plant defenses, particularly against plant pathogens^25^. For example, *B. bassiana* and *Metarhizium* spp. colonization was found to evoke microbe-associated molecular patterns, to trigger immunity and to induce several JA and SA signaling pathway genes^26,27^. Both signals, JA and SA, induce a broad spectrum of defensive responses such as the production of alkaloids and volatile organic compounds^28^. Even though an increase in JA levels should be negative for cabbage root flies, since the JA pathway is the main defense pathway against insect herbivores^29^, the fact that there is no significant increase in roots, which is where larvae feed, and where the highest amount of infective *M. brunneum* spores were found (data not presented here), the biological effect of this increase in JA in leaves may be negligible for larvae. However, since leaves produce the majority of the volatile profile that insects use to identify and orient towards their host plants^30^, an increase in the change of the volatile profile associate with up regulation of the JA pathway may explain why cabbage root fly females prefer FIP plants.

### Indirect effects of R-AEF on herbivorous insect behaviors

Insect attraction to host plants is modulated by a combination of factors, which in combination are honest signals of host quality. Chief among these are color that is often correlated with nutritional quality, and volatile profile, which is directly related to plant species, defenses and the presence or absence of herbivory^31–34^. R-AEF increased the attraction and oviposition of cabbage root flies, and presence of mycosed cabbage root fly larvae and pupae confirmed the entomopathogenic capability of this *Metarhizium* strain under field conditions^13^. Wind tunnel experiments showed that cabbage root fly females preferred FIPs based on the combination of volatile profile and color (Fig. 4). Consistent with other studies based on root fly species, once the volatiles have stimulated receptive females to look for a suitable host plant, host plant finding is governed by plant color stimuli^35–37^. It is virtually impossible to determine the relative effects of R-AEF inoculation on leaf reflectance, phytohormone composition, and volatile profiles, but the combination of host plant responses enabled R-AEF to manipulate/increase the likelihood of infecting cabbage root fly larvae.

Previous studies also indicated that the use of the *M. brunneum* F52 strain should be considered as a potential alternative against cabbage root fly control due to the high mortality observed in greenhouse and field studies^38^. Our study has shown that this strain may modulate host plant functional traits of white cabbage. The impact of this manipulation of the host plant’s traits may lead to greater susceptibility to herbivore attack by cabbage root flies. Thus, this attraction could increase dispersal of this R-AEF, as the cabbage root fly is also a host, one which disperse the R-AEF to new host plants, but at the same time it could lead to the loss of its host if strong herbivory occurs. The effect of R-AEF on the studied system may have a detrimental impact to either the plant or the herbivore, but it may be beneficial for the fungi overall.

## Conclusions

The findings presented in this study add to a growing body of literature describing highly complex tri-trophic interactions and manipulative strategies involving micro-organisms, host plants and insect herbivores. Based on a combination of laboratory and field studies, we demonstrated how an entomopathogenic fungus, *M. brunneum,* associated with roots of white cabbage plants significantly altered host plants traits used by cabbage root flies in their host plant selection. We also demonstrated that these host plant manipulations increased the likelihood of *M. brunneum* encountering and infecting cabbage root fly individuals. Thus, our study results support the hypothesis outlined in Fig. 1 and provide novel insight into the possible functional roles and strategies of entomopathogenic fungi.

## Material and methods

### Insect rearing

Cabbage root flies were reared in a climate-controlled chamber maintained at 16L:8D photoperiod and 22 ± 2 °C temperature regime at a constant 75% relative humidity. Cabbage root fly larvae were reared on turnip (*Brassica napus*) and cabbage root fly adults were fed a mixture of honey, dried yeast and milk powder as described by Nilsson *et al*.^39^. Cabbage root fly adults were sexed based on the morphology of the abdomen.

### Root-associated entomopathogenic fungal endophyte culture

The commercial product, Met52 Granular bioinsectide (Novozymes Biologicals Inc., Salem, VA), was chosen for field and choice experiments in 2014 and 2015. This product consists of dried living spores of the entomopathogenic fungus *M. anisopliae* Strain F52 on a grain matrix. The *M. anisopliae* Strain F52 was re-classified as *Metarhizium brunneum* (Petch), but the commercial product is registered as *M. anisopliae*. The identification of the fungus isolated from the commercial product Met52 was verified by observation of dark green colored cylindrical conidia formed in chains characteristic of *Metarhizium* when growing on agar media (N.V. Meyling, pers. comm.)^40^. Met2 was purchased in 10 kg sealed bags containing 2% active ingredient on a rice carrier with 9.0 × 10^8^ Colony Forming Units (CFU)/g of formulated product. This strain has been tested on cauliflower plants as host plants with positive results against cabbage root flies^13^.

Met52 was not commercially available in 2016, so we had to produce the granular formulation for experiments in 2016 and 2018. The methodology to produce *M. anisopliae* Strain F52 on rice and host plants and soil inoculation was assessed as described by Cachapa *et al.*^41^, but we used Jasmine rice in our experiments. Conidial viability was assessed as described by Rännbäck *et al.*^42^ through counting the proportion of germinated conidia. Viability data collected from each of the experiments had a mean value of 73.4%.

### Host plants and soil inoculation

White cabbage was used as a host plant. Host plant treatments included: (i) non-inoculated control plants (NIP) and (ii) root-associated entomopathogenic fungal (R-AEF)-inoculated plants (FIP). NIPs were prepared by sowing seeds of the host plant directly in peat substrate (Så—Kaktusjord, 11-5-18 NPK, Hasselfors Garden AB, Örebro, Sweden), while for preparation of FIP, Met52 Granular bioinsecticide was mixed thoroughly with the peat substrate. Two concentrations of FIP were adjusted, a low concentration of Met52 Granular at 1.8 kg/m^3^ (FIP-Low) and a high concentration at 2.7 kg/m^3^ (FIP-High). The low concentration was based on the application rate recommended for nursery planting mix of the commercial *M. brunneum* product, Met52 Granular.

In the field experiment, host plant seedlings were transplanted into field settings. For the rest of the experiments, host plant seedlings were transplanted into 3 L pots. Potting soil consisted of compost amended with Leca granules (Exclusiv Blom & Plantjord, Emmaljunga, Sweden) and fertilized with a slow-release fertilizer (Basacote Plus 3M, 16-8-12 NPK, coated with trace elements; Compo, Münster, Germany). Host plants for the greenhouse experiment were grown in a controlled chamber for 4 weeks at 20 ± 1 °C, 65% RH and in 16L:8D under natural light and 400 W high pressure sodium lamps. For the other experiments, the potted host plants grew under the same conditions than for the greenhouse experiment, with the exception of being under only artificial lighting. For all experiments, except for the field one, host plants were tested for four to five weeks after transplanting.

### Experiment 1: leaf reflectance

We sampled individual leaves from NIP (n = 16) and from FIP-High (n = 17) 30 days after soil inoculation with Met52 Granular at a high concentration at 2.7 kg/m^3^. Host plant leaves were sampled and subjected to hyperspectral imaging. Similar to previously published studies^43–47^, we used a hyperspectral push broom spectral camera (PIKA XC, Resonon Inc., Bozeman, MT, USA). The objective lens had a 35 mm focal length (maximum aperture of F1.4) and was optimized for the visible and NIR spectra. The main specifications of the spectral camera are as follows: interface, Firewire (IEEE 1394b); output, digital (14 bit); 240 spectral bands (from 379 to 1031 nm) by 1600 pixels (spatial); angular field of view of 7°; and spectral resolution of approximately 2.1 nm. All hyperspectral images were collected with artificial lighting from 15 W, 12 V LED light bulbs mounted in two angled rows, one on either side of the lens, with three bulbs in each row. A piece of white Teflon (K-Mac Plastics, MI, USA) was used for white calibration, and ‘relative reflectance’ refers to proportional reflectance compared with that obtained from Teflon. Consequently, relative reflectance values ranged from 0 to 1. Prior to analysis, we excluded the first and last nine of the original 240 spectral bands, and the remaining 222 spectral bands (from 408 to 1011 nm) were spectrally 3×-binned (averaged) into 74 spectral bands^43,47–49^.

### Experiment 2: phytohormone composition

Host plant material from NIP and from FIP-High were freshly cut, frozen in liquid nitrogen directly after cutting, enclosed in a foil envelope and shipped to the Laboratory of Growth Regulators at Palacký University and Institute of Experimental Botany AS CR (Olomouc, The Czech Republic) for analysis. Samples were stored at − 80 °C prior to phytohormone quantification according to the method described by Floková *et al*.^50^ Levels of jasmonic acid (JA), (+)-7-iso-jasmonoyl-l-isoleucine (JA-Ile), cis-12-oxo-phytodienoic acid (cis-OPDA), abscisic acid (ABA) and salicylic acid (SA) were measured on two sections of host plants: leaves and roots. Samples were homogenized, weighed (5 mg DW in duplicate) and extracted in 1 ml of 10% methanol together with a cocktail of stable isotope-labelled standards as follows: 10 pmol of [^2^H_6_]JA, [^2^H_2_]JA-Ile, and [^2^H_6_]ABA, 20 pmol of [^2^H_4_]SA and [^2^H_5_]OPDA (all from Olchemim Ltd, Czech Republic) per sample. The extracts were purified using Oasis HLB columns (30 mg/1 ml, Waters Corporation, Milford, MA, USA) and the eluent (80% MeOH) containing targeted analytes was gently evaporated to dryness under a stream of nitrogen. Phytohormone levels were determined by ultra-high performance liquid chromatography-electrospray tandem mass spectrometry (an Acquity UPLC I-Class System coupled to a Xevo TQ-S MS, all from Waters) using stable isotope-labelled internal standards as a reference to validate phytohormone determination. A total number of 31 root sections and 24 leaves of NIP and FIP-High were analyzed for hormone profiling. Phytohormone levels are given in pmol/g DW.

### Experiment 3: landing preference

A two-choice wind tunnel experiment was carried out at the Norwegian Institute of Bioeconomy Research (NIBIO). Technical specification of the wind tunnel^30,31^. The temperature and humidity inside the wind tunnel were 23.5 ± 0.5 °C and 63.9 ± 2.5%, respectively and light intensity in the different sections was recorded (Table S1). The rest of details of the protocol are described in Online Appendix 1. Cabbage root fly females used in wind tunnel experiments were 7 to 12 days old, corresponding to the first oviposition peak of this fly species^51^. After testing, cabbage root fly female fertility was assessed by the presence of mature eggs. The wind tunnel experiment was performed between 13:00 and 17:00 corresponding with the time when cabbage root fly females oviposit in the field^52^. Twelve flies (four vials containing three flies each, vials tested individually in the wind tunnel) were tested for the same host plant material (NIP vs FIP-High) and considered a replicate. New host plant material was provided for each replicate (12 cabbage root fly females). Five behavioral steps were recorded:

- Non-responders (movement to a location outside of the glass tube, but not passing 50 cm towards the source).
- Upwind orientation over at least half the length of the wind tunnel (less than 100 cm).
- Full-distance flight (between 100 and 150 cm).
- Close approach (passed 150 cm and approaching the host plant closely without landing).
- Landing on a predefined landing area (130 × 300 mm; see Online Appendix 1).

Landing responses by flies were assessed with either host plant volatiles only (volatile cues) or whole host plants (visual and volatile cues) as attractants. A total number of 228 cabbage root fly gravid females were tested in the wind tunnel for two-choice responses, and 41 cabbage root fly females out of the total did not respond to any stimuli.

### Experiment 4: field experiment

From May to October in both 2014 and 2015, an experiment was conducted on a 0.56 ha experimental field at the Alnarp campus of the Swedish University of Agricultural Sciences (Alnarp, Sweden) where a randomized complete block design was used with three blocks. In 2014, each block contained two plots of each treatment (NIP and FIP-Low) with a total number of 288 plants (144 plants for each treatment). In addition, two more plots of a third treatment (FIP-High) were included in 2015, with 96 plants for each of the treatments. A single plot was represented by individual cages W 3.0 × L 6.0 × H 1.50 m (Online Appendix 2) where we transplanted 32 host plant seedlings. About 2 weeks after transplantation of host plant seedlings, we performed sequential releases of cabbage root fly adults into the cages (Online Appendix 2). The experiment was terminated 14 weeks after transplanting host plants seedlings, and we examined root systems and stem bases of host plants for cabbage root fly larvae and pupae. Cabbage root fly larvae found in host plants were transferred individually to 30 ml medicine cups containing 3 ml of sterile sand (0.8–1.2 mm, Rådasand, Sweden) moistened with 400 µl of sterile tap water, fed turnip pieces (1.0 × 1.0 × 0.3 cm) and incubated at 20 °C in darkness. Cabbage root fly larvae and resulting pupae were monitored for mortality every week for 6 months. Dead cabbage root fly larvae were surface-sterilized in 5% sodium hypochlorite (Sigma-Aldrich, Sweden), rinsed in deionized water and incubated for *M. brunneum* sporulation as described by Rännbäck, *et al.*^42^ Cabbage root fly pupae were checked once a week to record hatching of cabbage root flies (F1 generation), or mycosis by *M. brunneum*. A small fraction (< 5%) of the cabbage root fly pupae did not hatch within three months and were dissected.

### Experiment 5: propensity of oviposition

Cages [W 93.0 × L 47.5 × H 47.4 cm (BugDorm-4F4590DH, MegaView Science Education Services Co., Ltd., Taiwan)] were placed inside a greenhouse (20 ± 1 °C, 65% RH and in 16L:8D) from August to September in 2014 and 2016. We performed two types of oviposition experiments repeated four times: (i) no-choice experiment with a single host plant of the two treatments (n = 24) (NIP or FIP-Low) and (ii) two-choice experiment (n = 48) with one host plant of each treatment (NIP vs FIP-Low and NIP vs FIP-High). The no-choice experiment was performed for the low concentration to test whether cabbage root fly females laid more eggs on the NIP or FIP-Low when they were individually offered to cabbage root fly females. In each experiment, a couple of 6–8 days old cabbage root fly adults was released into a cage, and oviposition was recorded after 48 h. Food and water were provided in the same way as in the insect rearing, and all experiments were started between 9 and 11 am.

### Statistical analysis

In Experiment 1, we averaged pixels representing leaf surface (omitted background) to obtain average leaf reflectance from individual leaves from both treatments. Subsequently, we performed analysis of variance of average leaf reflectance for each host plant treatment in 75 individual spectral bands. In Experiment 2, we used GLM to compare levels of each of the phytohormones [jasmonic acid, (+)-7-iso-jasmonoyl-l-isoleucine, cis-12-oxo-phytodienoic acid, abscisic acid and salicylic acid] in roots and leaves from NIP and FIP-High plants. To analyze landing in Experiment 3, we examined preference of the cabbage root flies using a two-tailed binomial test with the 95% confidence level. We used generalized linear models (GLM) with a negative binomial distribution to test overall number of cabbage root fly specimens in Experiment 4, including host plant treatment (NIP and FIP) and block as a fixed factors. Then, we used the Tukey’s test for multiple comparisons. Finally in Experiment 5, we analyzed the overall number of cabbage root fly eggs laid on host plants in two-choice and no-choice experiments using a generalized linear mixed models (GLMMs) with a Poisson distribution. Host plant treatment was a fixed effect and the position of the cage in the greenhouse and repetition as random effects. The overall overview of the study is described in Fig. 1.

We analyzed data using the statistical software R v. 3.4.3^53^ and RStudio v. 1.1.423^54^ with packages *lme4*^55^ and *MASS*^56^. Regarding data from experiments 2, 4 and 5, we checked fixed factors for significance with the Wald-test using the *car* package^57^.

## Supplementary Information

Supplementary Information 1.

Supplementary Information 2.
