# Supplementary material for: Root-associated entomopathogenic fungi manipulate host plants to attract herbivorous insects
Source: Sci Rep. 2020 Dec 30;10:22424. doi: 10.1038/s41598-020-80123-5 (PMC7773740; doi:10.1038/s41598-020-80123-5)
Supplement: Supplementary file 1 — Supplementary Information 1. [file 41598_2020_80123_MOESM1_ESM.docx]

**Appendix 1.** Technical specification of the wind tunnel and protocol details:

*Wind tunnel hardware* is described in detail by Aak, Knudsen and Soleng (2010) and Knudsen, Tasin, Aak and Thöming (2018).

*Wind tunnel protocol:*

The wind tunnel setup had to be adjusted beforehand for our own conditions. Then, wind speeds in the wind tunnel cabinet was adjusted to 30 cm s^-1^.

A group of three flies were placed on the platform in the wind tunnel. The cloth was removed from both sides of the glass vials to release the flies and the response of the three females was followed during 10 minutes counting from the first moment when a fly left the vial. After the ten minutes, the flies were recovered in individual vials marked according to their responses and kept in the -20 °C for 24 h. Afterwards, female abdomens were dissected with forceps and scalpel using a stereomicroscope in order to check the ovary development and egg production of the tested females. Total absence of eggs was used as criteria to exclude females´ responses from the dataset.

We divided the inner space of the wind tunnel in four well differentiated sections and five different types of behaviors were recorded based on these sections (Table S1)

Table S1: Sections in wind tunnel, corresponding behavioural response and description of the responses and light intensity measured in each section:

| **Section in wind tunnel** | **Behavioural response** | **Description** | **Light intensity (lux)** |
| --- | --- | --- | --- |
| Platform | No responder | Movement to a location outside of the glass tube, but not passing 50 cm towards the source | 950 |
| 50-100cm | Upwind orientation | Over at least half the length of the wind tunnel (less than 100 cm) | 1175 |
| 100-150cm | Full-distance flight | Between 100 and 150 cm | 1105 |
| Final section 150 cm to plant | Close approach | Passed 150 cm and approaching the plant closely without landing | 1015 |
|  | Landing | On one of the two plants  Inside one of the oval black paper lines | 1015 |

*Treatments*

In a wind tunnel two-choice study, we assessed both whole plants (visual and volatile cues) and plant volatiles only (volatile cues) as attractants:

Testing chemical and visual cues, entire potted plants were used. The two plants were separated by 50 cm and were placed on plataform in upwind end of the flight section of the wind tunnel. The canopies (the odor ad visual source) were located exactly at the same height that the take off area of the flies (30 cm above the ground). The plant itself was defined as the landing area for the flies.

Testing chemical cues only, entire potted plants were placed with the same set-up as in the test with chemical and visual cues, but the visual source of the plants was hiden between the filter housing of the wind tunnel and the flight section. This 30-cm-section was separated with a perforated metal grid on each side (Aak et al. 2010; Knudsen et al. 2018), which allows a constant wind flow and odour transport through the wind tunnel, but eliminted visual cues from the plant. Two black paper lines representing an oval perimeter of 130 × 300 mm were placed directly on the vertical grid wall and was used as the landing area for the flies.
