# Supplementary material for: Root-associated entomopathogenic fungi manipulate host plants to attract herbivorous insects
Source: Sci Rep. 2020 Dec 30;10:22424. doi: 10.1038/s41598-020-80123-5 (PMC7773740; doi:10.1038/s41598-020-80123-5)
Supplement: Supplementary file 2 — Supplementary Information 2. [file 41598_2020_80123_MOESM2_ESM.docx]

**Appendix 2.** Field experiment set up.


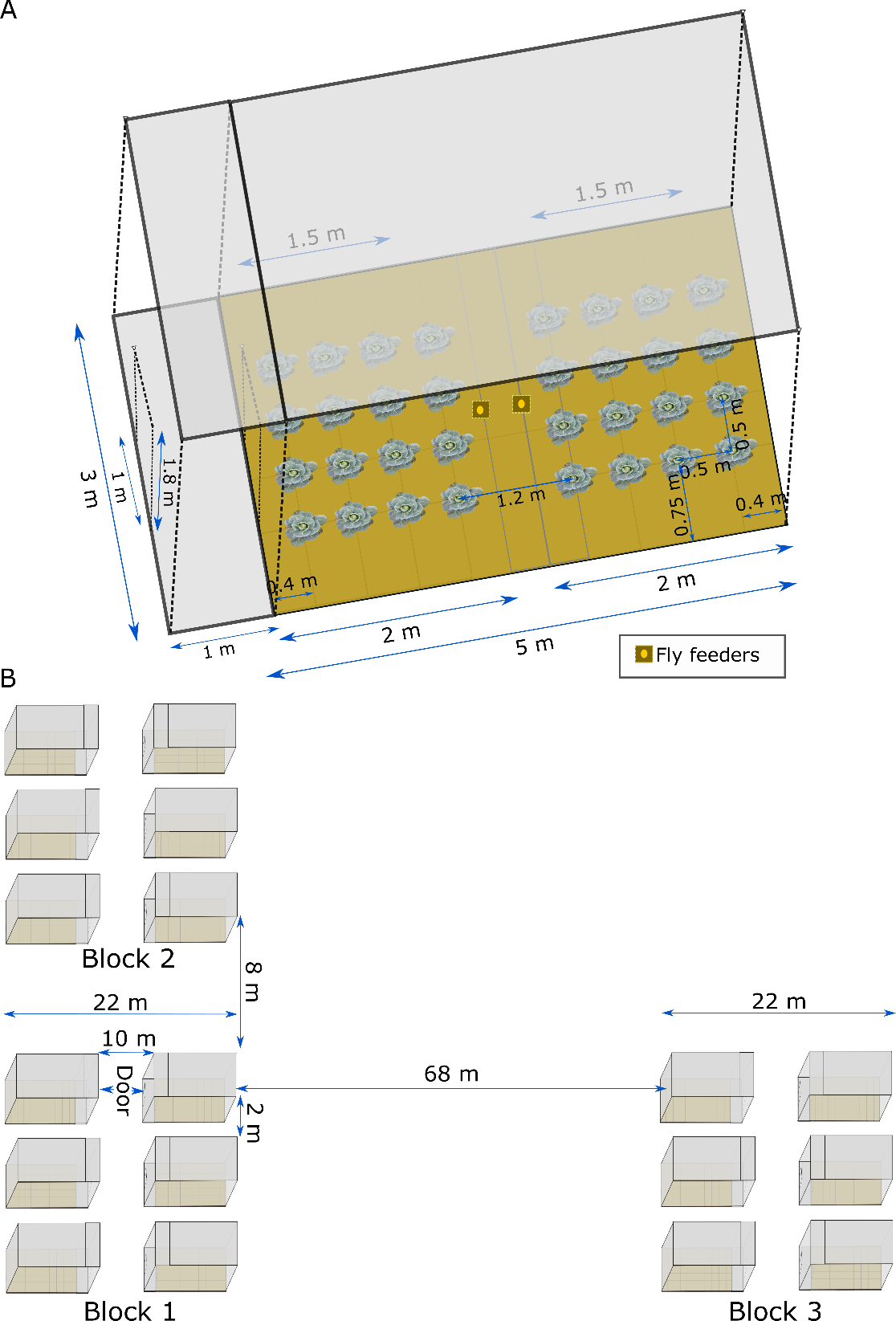


Figure S2. Diagram of the cage and field experiment set up. A) Cage scheme. Each cage had a biosafety entrance and a crop area where 32 cabbage plants were homogenously sown. Flies’ feeders were placed in the middle of the cages. Subsurface irrigation was supplied once a day. A rain sensor temporarily shut off the irrigation system so the system did not run during rain. Fertilization with chicken manure and weeding was done manually inside the cages B) Scheme of cages distribution: A randomized complete block design was used with three blocks. Each block was composed by 6 plots(=cages) equally organized. In 2014, two plots per block were used for non-fungal inoculated plants (NIP) and other two cages for fungal-inoculated plants with low concentration (FIP-Low). In 2015, two additional plots per treatment (NIP, FIP-Low and FIP-High) were used in each block.

Table S2. *D. radicum* release pattern in 2014 and 2015

| **Date of release** | **Male** | **Age (days)** | **Female** | **Age (days)** |
| --- | --- | --- | --- | --- |
| 18/08/2014 | 4 | 3 | 4 | 3 |
| 20/08/2014 | 20 | 2 | 20 | 6 |
| 21/08/2014 | 4 | 3 | 4 | 7 |
| 22/08/2014 | 6 | 2 | 6 | 4 |
| 25/08/2014 | 24 | 4 | 24 | 4 |
| Total 2014 | 58 |  | 58 |  |
| 07/07/2015 | 5 | 5 | 5 | 7 |
| 09/07/2015 | 3 |  | 3 |  |
| 13/07/2015 | 7 | 1 | 7 | 5 |
| 14/07/2015 | 5 | 1 | 5 | 4 |
| 15/07/2015 | 7 | 2 | 10 | 5 |
| 17/07/2015 | 10 | 1 | 7 | 4 |
| 20/07/2015 | 7 | 3 | 7 | 5 |
| 21/07/2015 | 6 | 1 | 6 | 4 |
| 24/07/2015 | 17 | 1 | 17 | 4 |
| 26/07/2015 | 7 | 1 | 7 | 3 |
| 27/07/2015 | 7 | 2 | 7 | 4 |
| 29/07/2015 | 14 | 1 | 14 | 4 |
| Total 2015 | 95 |  | 95 |  |
